# Edaravone Exerts Brain Protective Function by Reducing the Expression of AQP4, APP and Aβ Proteins

**DOI:** 10.1515/biol-2019-0074

**Published:** 2019-12-31

**Authors:** Haiyan Ren, Lijuan Ma, Xueli Gong, Chenbo Xu, Yuge Zhang, Meilei Ma, Kenichi Watanabe, Juan Wen

**Affiliations:** 1 Department of Pathology and Pathophysiology, Basic Medical College of Xinjiang Medical University, No. 393, Xinyi Road, Xinshi District, Urumqi 830011, P.R. China; 2 Laboratory of Electron Microscopy, Central Laboratory of Xinjiang Medical University, Urumqi 830011, P.R. China; 3 Department of Biochemistry, Basic Medical College of Xinjiang Medical University, Urumqi 830011, P.R. China; 4 Department of Clinical Pharmacology, Niigata University of Pharmacy and Applied Life Sciences, Niigata City 950-2181, Japan

**Keywords:** Cerebral ischemia-reperfusion injury, Aquaporin 4, β-amyloid precursor proteins, β-amyloid, Edaravone

## Abstract

This study aims to investigate the changes of aquaporin-4 (AQP4), β-amyloid precursor proteins (APP) and β-amyloid (Aβ) in brain tissues after cerebral ischemiareperfusion injury (CIRI), and evaluate the effect of edaravone. The Middle Cerebral Artery Occlusion was used to establish CIRI in rats. Rats were divided into control, model and edaravone groups. The neurological deficits in the model group were obvious and the neurological score increased compared to the control group, while the neurological deficits of the edaravone group were improved as the neurological score decreased compared to the model group. The number of pyramidel cells in the hippocampus of the model group was significantly decreased whereas edaravone could reverse this decrease. The model group had significantly higher levels of Aβ, APP and AQP4 than the control group and edaravone group, suggesting that they might be involved in the neuronal cell damage. Meanwhile, the increased AQP4 might enhance the permeability of cells, and thus cause cell damage and neurological deficit. Conclusively, edaravone could reduce brain edema, protect neuronal cells and improve the neurological impairment of rats possibly by decreasing the expression of Aβ, APP and AQP4. Therefore, edaravone may have the potential to treat neurodegenerative diseases (such as Alzheimer's disease).

## 1 Introduction

Stroke, as a major cause of disability, can reduce the mobility of more than half of the stroke survivors older than 65 [1]. After stoke, reactive oxygen species (ROS), especially free radicals, play an important role in brain injury [2]. The cerebral artery can be occluded by a thrombus and cellular metabolism is impaired. These can further lead to cytotoxic and vasogenic edema, and finally the ischemic brain tissue would be converted to hemorrhage [3, 4].

Aquaporin-4 (AQP4) is a dominating protein related to water-transportation in brain and has a wide distribution in brain tissues. AQP4 plays a role in super-acute ischemic brain edema [5]. Papadopoulos *et al*. [6] indicated that AQP4 deletion worsened cerebral edema and resulted in lower neurological score. The brain edema model can be established through middle cerebral artery occlusion (MCAO), and after MCAO, AQP4-deficient mice had lower neurobehavioral scores than normal control mice [7]. It is concluded that AQP4 clears the excess fluid accumulated in the extracellular area in vasogenic cerebral edema [7].

Alzheimer’s disease (AD) is featured by β-amyloid (Aβ) protein deposition in brain [8]. The incidence of AD is greatly increased by hypoxic injury caused by cerebral ischemia or stroke [9]. Consistently, a study shows that the risk of AD is significantly increased after cerebral ischemia [10]. Thus, it is necessary to evaluate the overlapped mechanisms of these two diseases [11]. Aβ is generated by protein hydrolysis, in which β-amyloid precursor proteins (APP) are successively cleaved by β- and γ-secretases [12]. Cerebral ischemia briefly up-regulates APP and leads to its proteolytic products accumulating in the cortex region close to the ischemic lesion and alba [13].

It has been found that edaravone (MCI-186, 3-methyl-1-phenyl-2-pyrazolin-5-one), acting as a free radical scavenger, could protect cell membranes against oxidative stress, thus protecting the cerebrovascular endothelial cells and neurons [14]. Unlike other free radical scavengers, the molecular weight of edaravone is low. And, it is water-and lipid-soluble and could cross the blood-brain barrier rapidly [15]. Edaravone may protect neurons through the following mechanisms: (1) suppressing OH-dependent and OH-independent lipid peroxidation via quenching hydroxyl radical (OH); (2) inhibiting peroxidation systems and (3) repressing lip-oxygenase pathways and non-enzymatic lipid peroxidation [16]. Edaravone can improve ischemia and neuron death, thus alleviating cerebral edema and neurological deficits [17, 18].

Cerebral ischemia-reperfusion injury (CIRI) was induc ed in rats through MCAO. The expression of AQP4, APP and Aβ in CIRI was determined. The role and mechanisms of edaravone in CIRI were investigated.

## 2 Materials and Methods

### 2.1 Animals

Adult male Sprague Dawley (SD) rats (290±10 g; 8-10 weeks old) were from Vital River Laboratory Animal Technology Co., Ltd. (Beijing, China). They were kept in a standard condition. As previously described [19], a total of 90 rats were used and all numbered in order of body weight and block randomized first. Then simple randomization within each block was performed. Rats were randomly assigned into the control group (n=30), model group (n=30), and edaravone group (n=30). After the random grouping, experiments were performed according to the requirements of blind method, and the experimenters and analysts did not known about the grouping.

**Ethical approval:** The research related to animals use has been complied with all the relevant national regulations and institutional policies for the care and use of animals. This study was approved by the ethics review board of the First Affiliated Hospital of Xinjiang Medical University.

### 2.2 MCAO model establishment

For anesthesia, rats received 400 mg/kg of chloral hydrate intraperitoneally (i.p.). For the model group, after exposing artery, MCAO monofilament (2838-A4, Beijing Cinontech Corporation, Beijing, China) was placed into the internal carotid artery and the middle cerebral artery was occluded [20]. The occlusion lasted for 2 h, and then the reperfusion was performed for about 46 h. In the edaravone group, edaravone (6 mg/kg body weight) was infused via tail vein at 15 min before reperfusion. In the control group, rats received the same surgical procedures but without occlusion or reperfusion.

After 48 h of cerebral ischemia and reperfusion, the behavioral tests of grip strength and locomotor activity was respectively performed. Immediately after behavioral tests, the rats were killed. Their brains were collected and used for measuring the infarct area and HE staining. Brain homogenate was prepared and biochemical tests were conducted.

### 2.3 Nerve symptom scores

According to the Zea Longa’s level 5 evaluation method [21], the nerve symptom scores in 48 h after surgery were respectively assessed. Criteria: 0 represents no obvious neurologic symptoms; 1 represents failing to fully extend on the left paw; 2 represents rotating to the left; 3 represents walking to the left; 4 represents unable to walk.

### 2.4 Measurement of infarct size

The brain tissue was removed and cut into coronal sections (2.0-mm-thick). Then, the brain sections were stained with 2% TTC (2, 3, 5-triphenyl tetrazolium chloride) for 30 min at 37 °C. After that, the sections were fixed with 10% formalin for 24 h [22]. Normal tissues were stained reddish brown in color, whereas the infarcted areas were in dull yellow color. Based on the color difference, the infarct size was calculated. The investigator measuring the infarct size was blinded to the grouping.

### 2.5 Haematoxylin-Eosin (HE) staining

HE staining was performed according to a routine procedure. Briefly, after 48 h of ischemia-reperfusion, rats of each group were anesthetized by chloral hydrate (i.p.). Then, intracardiac perfusion of 4% paraformaldehyde solution and phosphate buffer (0.1 mol/L, pH=7.4) was performed for fixation. After fixation for 1 week, brain tissues were cut into coronal continuous sections (4 μm) and then the sections were dewaxed, dehydrated, stained and sealed. Morphological changes of neurons in the hippocampus were observed with microscope (Nikon, Japan) at 400× magnification. The number of pyramidal cells in the hippocampal CA1 region in two consecutive views was counted under high magnification (400×). The average cell number of the two views was used to represent the pyramidal cell number of the slice, and the mean of three slices was considered as the pyramidal cell number of the group.

### 2.6 Immunohistochemistry

The antigens in the brain tissue slices were first repaired by microwave in 0.01 mol/L citrate buffer, and then respectively incubated with primary antibody (AQP4 antibody, 1:100, Santa Cruz Biotechnology, Inc., Santa Cruz, CA; APP antibody,1:100, Abcam, Camb., UK; Aβ antibody,1:50, Abcam, Camb., UK) at 4°C for 40 h. After washing, horseradish peroxidase (HRP)-conjugated secondary antibody (Neobioscience, Shenzhen, China) was added and incubated at 37°C for 1 h. After washing, DAB (diaminobenzidine/peroxide) was used for color development. Then, for counterstaining, sections were incubated in hematoxylin for 5 min. Finally, 5 fields of the right cortex and hippocampal areas of each group were observed under an optical microscope (Nikon, Japan). The integral optical density (IOD) of AQP4, APP and Aβ were analyzed with the image acquisition system of NIS-Elements Basic Research (Nikon, Japan).

### 2.7 Western Blot

The protein was isolated from brain homogenate and protein concentration was measured. Proteins were subjected to 12% SDS-PAGE and transferred to PVDF membranes. After blocking for 1 h in 5% non-fat milk , the membranes were incubated with primary antibodies of anti-AQP4 (1:20000), anti-APP (1:20000), anti- Aβ (1:1000) and anti-β-actin (1: 2000) rabbit antibody overnight at 4°C. After washing, goat anti-rabbit IgG-HRP (1:20000) was added and incubated for 2 h at room temperature. The ECL detection system (Merck-Millipore, Billeric, MA) was used for visualizing immunoreactive bands and the band intensity was measured using Image J.

### 2.8 Statistical Analysis

SPSS 11.5 (SPSS, Chicago, IL, USA) was used. One-way analysis of variance was used for the comparison among groups, and SNK-q test was used for pairwise comparison when the variance analysis results showed significant differences. *P* less than 0.05 was considered to have statistically significant differences.

## 3 Results

### 3.1 Edaravone decreases the neurological scores of MCAO rats

**Figure 1:**
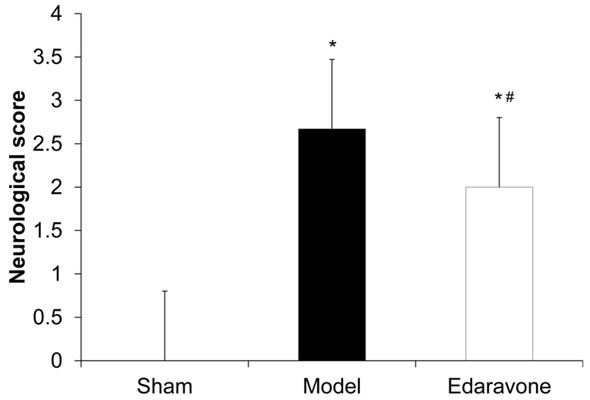
The effect of edaravone on the neurological impairment. The neurological scores of MCAO rats were analyzed. N=10. Compared to the sham group, *P < 0. 05. Compared to the model group, ^#^ *P* < 0. 05.

To determine the reliability of MACO, the neurological scores were evaluated. There was no obvious neurological deficits in the control group, while the model group showed significantly increased neurological scores (*P* < 0.05). The edaravone group showed significantly reduced neurological scores compared to the Model group (Fig. 1, *P* < 0.05). This indicates that edaravone can reduce the neurological score of rats after CIRI and improve the symptoms of nerve defects.

### 3.2 Edaravone reduces the cerebral infarction area in MCAO rats

**Figure 2:**
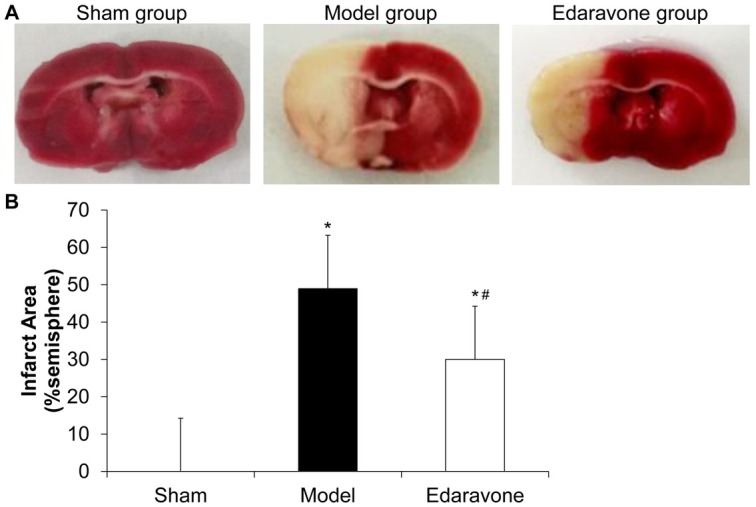
The effect of edaravone on the cerebral infarct size. (A) At 48 h after reperfusion, coronal sections of MCAO rats from sham, model and edaravone groups were obtained. TTC staining was conducted. (B) The infarct size was measured using Image J software. Infarct size was compared between the model group and edaravone group. N=10. Compared to the sham group, *P < 0. 05. Compared to the model group, ^#^ *P* < 0. 05.

To determine whether the MCAO method was reliable, TTC staining was performed. The normal cerebral area was stained red while the infarct area was un-stained and in white color. As shown in Fig. 2A, no cerebral infarction was observed in the control group. There was severe infarction in the model rats. Compared with the model rats, the edaravone-treated rats had less severe infarction (Fig. 2A). Statistically, the edaravone group significantly reduced the infarct size compared to the model group (49% *vs*. 30%, Fig. 2B, *P* < 0.05). This indicates that the cerebral ischemia-reperfusion model is successfully proven , and edaravone can reduce the cerebral infarction area after cerebral ischemia-reperfusion and reduce brain injury.

### 3.3 The effect of edaravone on the number of pyramidal cells of MCAO rats

**Figure 3:**
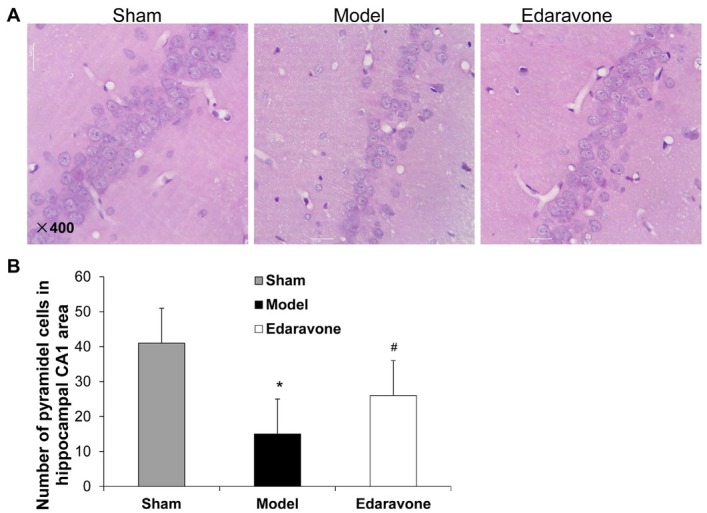
The effect of edaravone on the pathological changes in MCAO rats. Brain tissues were collected from sham, model and edaravone groups at 48 h after reperfusion. HE staining was performed to evaluate the pathological changes. (A) Representative HE staining results (400×). (B) The number of pyramidal cells in hippocampal CA1 region. N=10. Compared to the sham group, *P < 0. 05. Compared to the model group, ^#^ *P* < 0. 05.

To determine the pathological changes after cerebral ischemia-reperfusion, HE staining was performed. As shown in Fig. 3A, densely pyramidal cells were observed in hippocampal CA1 region of the control rats. By contrast, the model group rats showed scattered neurons, and the number of pyramidal cells significantly lowered (*P* < 0. 05). Compared to the model group, the number of pyramidal cells in the edaravone group increased significantly (*P* < 0. 05) (Fig. 3B). This indicates that the number of pyramidal cells in the hippocampal CA1 region of rats is reduced after cerebral ischemia-reperfusion, and edaravone could alleviate this reduction.

### 3.4 The effect of edaravone on AQP4, APP and Aβ levels in MCAO rats

**Figure 4:**
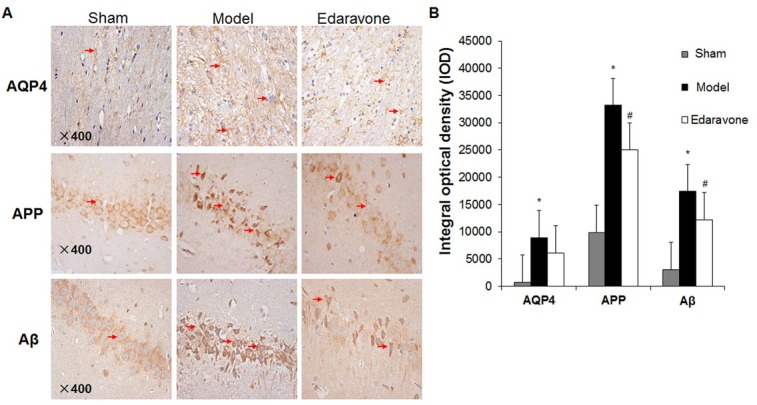
The effects of edaravone on the levels of AQP4, APP and Aβ in the infarct area of MCAO rats. Brain tissues were collected from sham, model and edaravone groups at 48 h after reperfusion. Expressions of AQP4, APP and Aβ were measured with immunohistochemistry. (A) Representative immunohistochemistry results (400×). (B) Number of AQP4, APP and Aβ-positive cells in the MCAO rats. The intensities of anti-AQP4, APP and Aβ immunoreactivity in the infarct areas were represented by IOD, which were calculated using Image J. Compared to the sham group, *P < 0.05. The arrows indicate cells with positive AQP4, APP and Aβ expression, respectively. N=10. Compared to the model group, ^#^ *P* < 0.05.

In order to evaluate the effects of edaravone on AQP4, APP and Aβ levels in MCAO rats, immunohistochemical analysis was performed. Comparing to the control group, the immunoreactivity of AQP4 was enhanced in the right cortex region of the rats, and the immunoreactivity of APP and Aβ were enhanced in the right hippocampal CA1 region of the rats in the model group. However, their immunoreactivity significantly decreased in the edaravone group (Fig. 4A). Additionally, the IODs of AQP4, APP and Aβ were evaluated using Image J. As shown in Fig. 4B, IOD in the infarct area of the edaravone group was significantly reduced compared to that of the control

group (*P* < 0.05).

**Figure 5:**
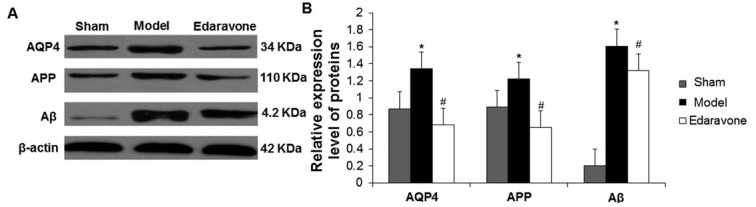
The effects of edaravone on the expression of AQP4, APP and Aβ in the infarct area of MCAO rats. Brain tissues were collected from control, model and edaravone groups at 48 h after reperfusion. Expression levels of AQP4, APP and Aβ proteins were detected with Western blot. (A) Representative Western blot images. (B) Relative protein expression levels. N=10. Compared to the sham group, *P < 0.05. Compared to the model group, ^#^*P* < 0.05.

Moreover, the level of AQP4 in the right cortex, and the levels of APP and Aβ in the right hippocampus and cortex were examined by Western blot. The levels of these proteins were up-regulated in the model group compared to that in the control group, whereas they were decreased in the edaravone group compared to the model group (Fig. 5A). Statistically, the AQP4, APP and Aβ levels were significantly higher in the model group than those in the control group (*P*< 0.05) (Fig. 5B). These proteins showed significantly lower levels in the edaravone group than those in the model group (*P*< 0.05) (Fig. 5B). Although the levels of AQP4 and APP in the edaravone group were lower than those in the control group, which was different from the trends in the immunohistochemical experiment, the difference had no statistical significance (*P* > 0.05). This indicates that the AQP4, APP and Aβ levels in the brain increase after CIRI, and they might cause damage to the neurons in the hippocampus of the brain. However, edaravone could reduce neuron damage by inhibiting the expression of these three proteins.

## 4 Discussion

It has been shown that AQP4 gene knockout can alter the pathophysiological processes of neurological diseases, such as stroke [23] and AD [24]. Aβ cascade is vital in AD etiology. Aβ accumulation in the brain parenchyma may contribute to the occurrence of sporadic AD [25]. In order to investigate whether there are some common pathological links in stroke and AD, we used the suture method to block the middle cerebral artery and simulate stroke, and observed the expressions of the characteristic protein Aβ, its precursor APP and AQP4 after CIRI. The results found that the three proteins showed similar increasing trends after CIRI. This indicates that they are all involved in neuronal cell injury following cerebral ischemia-reperfusion. These results also imply that stroke and AD may not be two distinct diseases and may influence each other. Study on the mechanism of toxicity of Aβ finds that astrocytes, which have the largest number in mammalian brains, participate in brain homeostasis of Aβ transport and clearance [26]. Reactive astrogliosis adjacent to amyloid plaques is considered as the feature of AD [22]. Aβ exerts toxic effects on neurons and astrocytes [27]. The Aβ formation can be prevented by cleavage of APP by α-secretase [28]. In addition, more ROS may be produced by mitochondria after interaction with Aβ [29]. Free radical stress participates in AD pathogenesis [30], which can further promote Aβ accumulation and enhance the pathogenic cascade [31]. The above results indicate that the toxic effect of Aβ may be induced by increasing free radicals after CIRI.

Morphological changes of astrocytes induced by Aβ1-42 are significantly decreased if AQP4 gene is absent, indicating that AQP4 might participate astrocyte activation. Consistently, AQP4 deletion impairs astrocyte migration, reactive astrogliosis, and glial scar formation during cortical stab injury [32]. The effects of Aβ1-42 on cultured astrocytes may be mediated by AQP4, as AQP4 deficiency decreased Aβ uptake, and in turn reduced astrocyte activity and apoptosis [33]. The above studies indicate that Aβ and AQP4 have consistent changing trend after CIRI.

Edaravone is able to reduce AQP4 protein levels in the brain infarct area in MCAO rats [19]. Moreover, edaravone inhibited Aβ via increasing α-secretase-formed fragments and decreasing β-secretase-formed fragments dose-dependently in transfected SY5Y-APP695swe cells [34]. The decline of Aβ levels might be caused by the following mechanisms: (1) reducing APP expression; (2) increasing nonamyloidogenic pathway, or decreasing amyloidogenic pathway, or both; (3) increasing the activity of Aβ-degrading enzymes or enzyme expression [34]. The possible mechanism underlying the effects of edaravone on these three proteins may be as follows. First, Aβ acts on neurons and induces mitochondria to produce more ROS, while ROS can promote the aggregation of Aβ to a greater extent, resulting in toxic effects. Edaravone, as a free radical scavenger, may down-regulate Aβ expression by clearing ROS. In addition, *in vitro* results show that edaravone may increase α-secretase fragment formation and reduce β-secretase fragment formation in a dose-dependent manner, thereby inhibiting Aβ production [34]. Second, in the process of CIRI, free radicals can activate proteases and phospholipases, causing lipid peroxidation in the cell membrane and capillaries and thereby undermining the blood-brain barrier and up-regulating AQP4 expression. Edaravone inhibits AQP4 expression by scavenging free radicals. In-depth functional study should be performed to demonstrate the detailed mechanism of edaravone in brain protection.

This study is limited in that no functional assay was performed. The functional assays, such as the effect of edavarone on proliferation and production of ROS, will be performed in future studies.

In summary, the findings demonstrate that edaravone has therapeutic potential in AD or other neurodegenerative disorders, in which free radical is involved. However, future studies on the detailed mechanism underlying the suppressive effect of edaravone on AQP4, APP and Aβ are warranted.
